# Integrated Metabolomics and Transcriptomics Analysis Reveals New Insights into Triterpene Biosynthesis in *Rosa rugosa*

**DOI:** 10.3390/plants13121600

**Published:** 2024-06-08

**Authors:** Guo Wei, Yang Xu, Pengqing Wang, Hammad Hussain, Yudie Chen, Yuqing Shi, Kaikai Zhu, Mengjuan Bai, Yong Xu, Jianwen Wang, Liguo Feng

**Affiliations:** 1 College of Horticulture and Landscape Architecture, Yangzhou University, Yangzhou 225009, China; gwei@yzu.edu.cn (G.W.); estherxu_2023@163.com (Y.X.); wpq19980127@163.com (P.W.); hussainhammad788@gmail.com (H.H.); yudiechen1130@163.com (Y.C.); mz120221400@stu.yzu.edu.cn (Y.S.); 008335@yzu.edu.cn (M.B.); 007047@yzu.edu.cn (Y.X.); 007156@yzu.edu.cn (J.W.); 2 Co-Innovation Center for Sustainable Forestry in Southern China, Nanjing Forestry University, Nanjing 210037, China; kkzhu@njfu.edu.cn

**Keywords:** *Rosa rugosa*, triterpenoids, oxidosqualene cyclase

## Abstract

*Rosa rugosa* is highly regarded for its aesthetic and therapeutic qualities. In particular, *R. rugosa*’s flowers are known to produce essential oils containing a mixture of volatile terpenes, phenylpropanoids, and other compounds. Despite this, extensive research exists on volatile terpenes in flowers, while the knowledge of non-volatile terpenes in distinct tissues is still limited. Using UPLC–ESI–MS/MS, a comprehensive analysis of the terpene metabolites in five different tissues of *R. rugosa* was conducted. These metabolites accumulated in distinct tissues, and the majority of them were triterpenoids. Transcriptome data were collected from five tissues using RNA-seq. Transcriptomics and metabolomics were utilized to evaluate the triterpene biosynthesis pathway, resulting in new insights into its regulation and biosynthesis. The *RrOSC10* was identified as a key enzyme in converting 2,3-oxidosqualene into α-amyrin, potentially contributing to the triterpene biosynthesis pathway. Furthermore, the expression of the *RrOSC10* gene was upregulated by salinity for 0.5 h and 1 h, with subsequent downregulation at 2 h. This study lays a foundation for future research on the biosynthesis and accumulation of triterpenes in *R. rugosa*.

## 1. Introduction

*Rosa rugosa* Thunb is a flowering shrub species native to eastern Asia that belongs to the Rosaceae family. It is known for its numerous attractive features, adaptability, and traditional medicinal application, which have led to its worldwide recognition [1]. *R. rugosa* has a wide range of applications, including the development of teas, juices, and wines [2]. Notably, their significant content includes a variety of biologically active compounds, such as anthocyanins, terpenoids, procyanidins, and proanthocyanidins [3,4,5]. Particularly, *R. rugosa* flowers have a pleasant aroma and are utilized to produce essential oil, which typically contains volatile mono-sesquiterpenes and phenylpropanoids/benzenoids [6]. A comprehensive understanding of the terpenoid composition of *R. rugosa* is needed to highlight its medicinal importance [7]. For instance, these plants have been utilized in traditional herbal medicine to treat various conditions, including stomach issues, pain, and inflammatory diseases [8]. While numerous studies have focused on the regulation and biosynthesis of volatile terpenoids in flowers [9,10,11,12], this study shifts the focus from volatile to non-volatile terpenes, which are present in different plant tissues, to identify previously undiscovered compounds that play a pivotal role in the medicinal properties of plants [13,14]. 

Terpenoids represent the most abundant and structurally diverse class of secondary metabolites detected in plants, with over 80,000 reported compounds [15]. They serve various functions in biological processes, such as attracting beneficial allies or repelling natural enemies [16]. Based on their structures, they can be classified into monoterpenes (C_10_), sesquiterpenes (C_15_), diterpenes (C_20_), sesterterpenes (C_25_), triterpenes (C_30_), etc. [17,18,19]. While the mevalonate (MVA) pathway, located in the cytoplasm, synthesizes precursor molecules essential for sesquiterpene and triterpene biosynthesis, the 2-C-methyl-d-erythritol 4-phosphate (MEP) pathway, situated in plastids, generates precursor compounds responsible for monoterpene, diterpene, and sesterterpene biosynthesis [20]. Among plant metabolites, triterpenes are recognized for their significant chemical diversity, with almost 200 unique skeletons identified [21]. Triterpenes display a variety of properties, which may have either positive or negative effects on human health, as well as antinutritional, sweet, or bitter effects [22,23]. Triterpenes, including sterols, are synthesized through 30-carbon intermediate 2,3-oxidosqualene, cyclized by various members of the oxidosqualene cyclase (OSC) family [24,25]. In plants, 2,3-oxidosqualene can transform into cycloartenol with the chair–boat–chair (CBC) conformation. Alternatively, this substrate can also take on the chair-chair-chair conformation (CCC) prior to cyclization, leading to a vast array of skeletal variations of triterpenes [26]. In recent years, OSCs have played critical roles in regulating plant growth and development and the response to environmental stresses in various plant species [27,28]. Generally, OSC enzymes facilitate the biosynthesis of triterpene scaffolds, such as *α*-amyrin, *β*-amyrin, and lupeol, using the substrate 2,3-oxidosqualene [29]. Nevertheless, the insufficient disclosure of OSCs in *R. rugosa* has hindered the progress of triterpene research in roses [30]. 

The *R. rugosa* plant is renowned for its medicinal and edible properties, along with its production of a wide variety of secondary metabolites. Nevertheless, there is still a lack of understanding regarding its non-volatile terpenoids and the molecular process of triterpene biosynthesis in the plant. Non-volatile terpenoids, particularly triterpenoids, play an important role in the ecological interactions of plants and hold significant relevance in their medicinal applications, which have captivated human interest for centuries. For instance, triterpene compounds, such as tormentic acid and rosamultin, are mainly present in the roots of *R. rugosa*. These compounds exhibit anti-inflammatory and anti-HIV protease activities and protect cardiac muscle cells against oxidative stress and cellular apoptosis [31]. The biosynthesis of rosamultin begins with “*α*-amyrin” and undergoes a three-step process catalyzed by cytochrome P450 monooxygenases (CYP450s) enzymes. Subsequently, a glycosylation reaction is carried out by uridine diphosphate glycosyltransferase (UGT) [30]. CYP450s exhibit several biocatalytic activities, including oxidation, epoxidation, hydroxylation, and demethylation [32]. In the triterpene biosynthesis, the CYP716A enzyme is capable of catalyzing *α*-amyrin to ursolic acid, lupeol to betulic acid, and *β*-amyrin to oleanolic acid [33,34]. The CYP716C enzyme can convert ursolic acid to corosolic acid and oleanolic acid to maslinic acid [35].

This study aimed to address the lack of knowledge about non-volatile terpenoid biosynthesis in *R. rugosa* through two primary objectives. The first objective was to comprehensively determine the presence of non-volatile terpenoids in different tissues of *R. rugosa*. The ultra-performance liquid chromatography–electrospray ion source–mass spectrometry/mass spectrometry (UPLC–ESI–MS/MS) system was utilized to analyze the intricate chemical profiles of non-volatile terpenoids in *R. rugosa*. The second objective was to investigate the genetic basis of triterpene formation in *R. rugosa* by identifying the specific *OSC* genes responsible for triterpene formation using the integration of metabolomics and transcriptomics approaches. To the best of our knowledge, this is the first report integrating metabolomics and transcriptomics data in analyzing different rose tissues, elucidating the accumulation patterns of triterpenoid compounds, and uncovering the molecular mechanisms underlying their formation.

## 2. Results

### 2.1. The Accumulation of Non-Volatile Terpenoids across Various Tissues of R. rugosa

**Figure 1:**
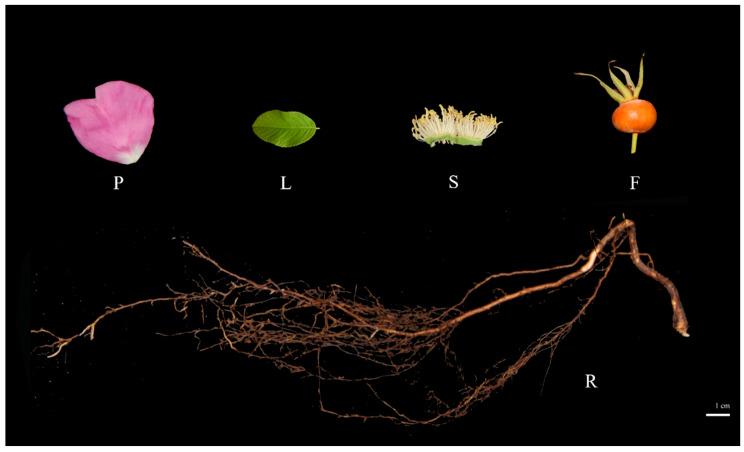
Five tissues of *R. rugosa*. P, petals. L, leaves. S, stamens. R, roots. F, fruits. Bars = 1 cm.

Samples of petals (P), leaves (L), stamens (S), roots (R), and fruits (F) were collected to analyze the variation in the non-volatile terpenoids using the UPLC–ESI–MS/MS system (Figure 1) (Supplementary Figure S1). In this study, the accuracy of expression analyses was validated by the Pearson correlation coefficient (PCC), which showed a significant correlation among the three replicated tissues (Supplementary Figure S2). A total of 74 terpenoids were identified and classified into the following three groups: 6 monoterpenoid derivatives, 5 sesquiterpenoid derivatives, and 63 triterpenes and triterpene saponins (Supplementary Figure S3A) (Supplementary Table S1). A principal component analysis (PCA) showed that the biological replicates had similar metabolite profiles, and the first two principal components accounted for 41.23% (PC1) and 22.94% (PC2), respectively. The non-volatile terpenoids were found to accumulate in both the fruit and root tissues. Similarly, a high similarity was observed in the accumulation of non-volatile terpenoids in the petals, leaves, and stamen tissues (Supplementary Figure S3B). 

**Figure 2:**
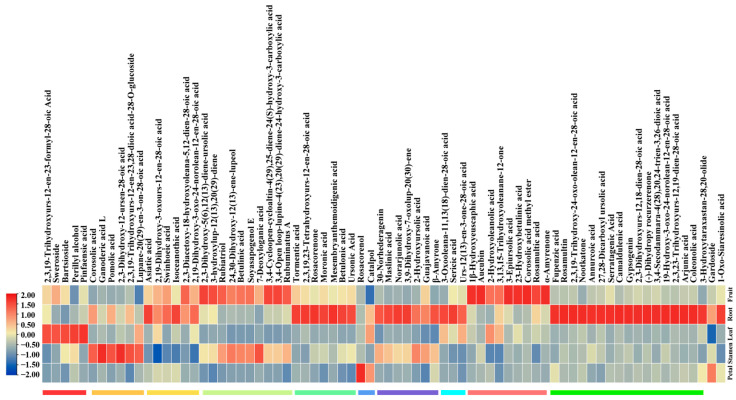
Metabolomic analysis of different tissues. Clustering heatmap of differential metabolites. Colors indicate distinct subclasses and demonstrate similar trends in metabolites.

The accumulation patterns of non-volatile terpenoids were analyzed through K-means and clustering heatmap analyses, leading to the identification of ten subclasses, labeled 1 to 10 (Figure 2) (Supplementary Figure S3C). The tissue-specific accumulation patterns of terpenoids were analyzed. Subclass 6 showed the highest levels of two non-volatile terpenoids in petals, while subclass 2 exhibited the highest levels of six non-volatile terpenoids in stamens. Subclass 1 exhibited the highest levels of five non-volatile terpenoids in leaves, whereas the roots contained the highest levels of non-volatile terpenoids from subclasses 3, 5, 7, 8, and 10. The highest levels of non-volatile terpenoids from subclasses 4 and 9 were found in fruits. Analyzing the tissue-specific patterns of terpenoids helped to identify potential genes associated with terpenoid biosynthetic pathways through metabolite–gene correlation analysis. It is noteworthy that triterpenoids were predominantly synthesized in roots and fruits.

**Figure 3:**
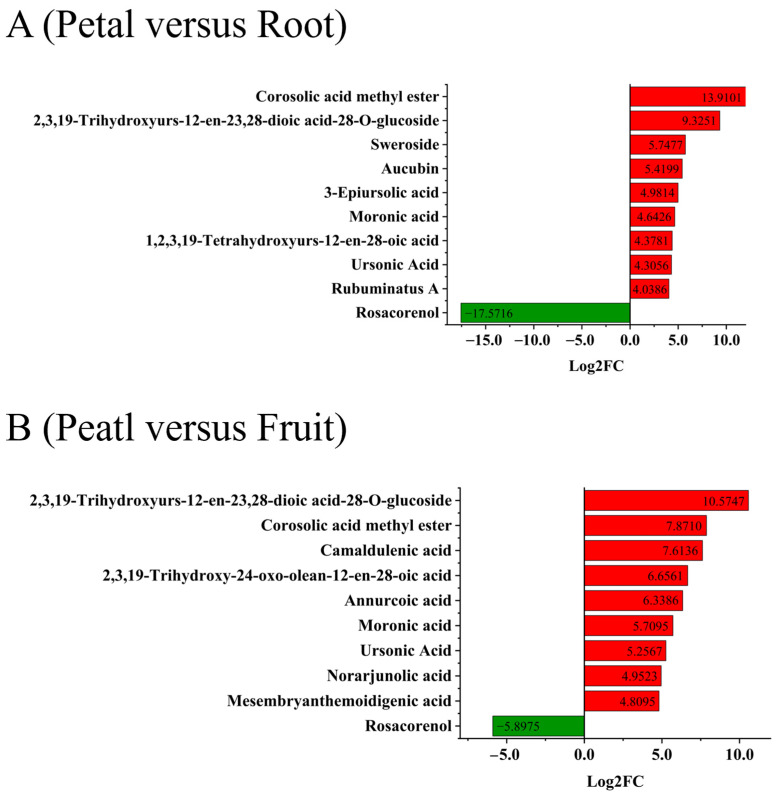
Differentially accumulated metabolites (DAMs) between tissues. (**A**) Top 10 metabolites in the comparison between petals and roots. (**B**) Top 10 metabolites in the comparison between petals and fruits. Red represents an increase in metabolite content, and green represents a decrease.

The analysis of differentially accumulated metabolites (DAMs) revealed a significant difference between the petals and roots. Specifically, 9 DAMs showed significant upregulation in the roots, while only one DAM (rosacorenol) displayed significant downregulation in the petals (Figure 3A). Interestingly, among these nine compounds, corosolic acid methyl ester, ursonic acid, 3-epiursolic acid, and rosamultin are likely derivatives of *α*-amyrin. Similarly, 9 DAMs were significantly upregulated in fruits compared to petals, while only one DAM (rosacorenol) showed significant downregulation in petals (Figure 3B). These compounds, such as corosolic acid methyl ester and ursonic acid, were also upregulated in fruits, reflecting their levels in roots.

### 2.2. Transcriptome Profiling across Different Tissues of R. rugosa

Transcriptome data were obtained from the five different tissues of *R. rugosa* using the RNA-seq method to understand the biosynthetic pathways of terpenoids comprehensively. These tissues are comprised of petals, leaves, stamens, roots, and fruit tissue. The average data generation was 12.23, 8.12, 9.64, 9.63, and 9.10 GB for the respective tissues (Table 1). Moreover, the average number of clean reads was 64,953,322 bp, with a minimal error rate of 0.03%. The quality scores, Q20 (%) and Q30 (%), ranged from 97.71 to 97.90 and 93.43 to 93.86, respectively. The GC content ranged from 46.52% to 47.46%. In total, the expression patterns of 29,758 genes are summarized in Supplementary Table S2.

**Figure 4:**
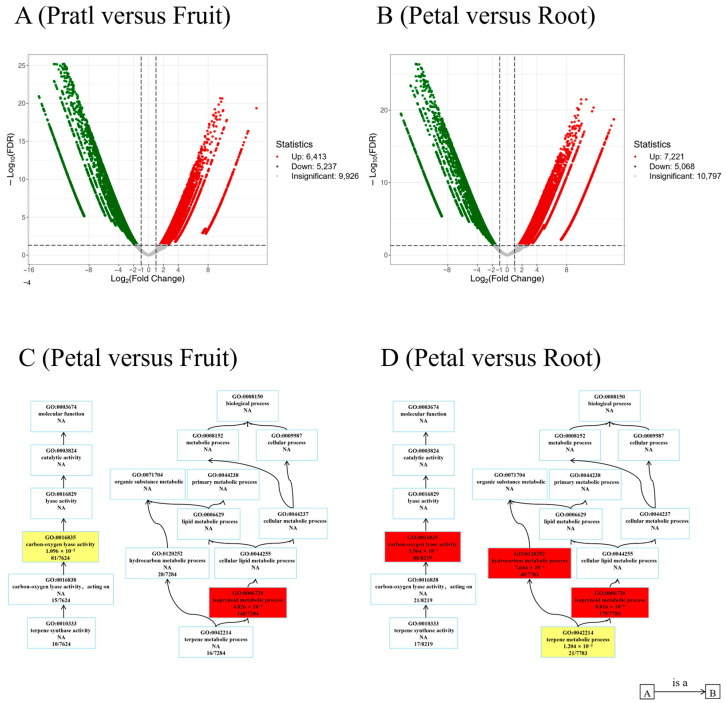
Differential expression gene (DEG) analysis between tissues. (**A**) Volcano plot displaying DEGs of petal and fruit tissues. (**B**) Volcano plot presenting DEGs of petal and root tissues. The x-axis shows the fold change in gene expression, and the y-axis represents the significance level of the DEG. (**C**) Directed acyclic graph (DAG) portraying gene ontology (GO) enrichment of DEGs from petal and fruit tissues. (**D**) DAG illustrating GO enrichment of DEGs from petal and root tissues.

DEGs between each tissue were obtained. Non-volatile terpenoids accumulated significantly in the fruit and root tissues, while petals, leaves, and stamen tissues showed high similarity. A further analysis was conducted to compare the characteristics of the petals with the fruit and the petals with the roots. Two sets of comparisons revealed varying quantities of DEGs. Compared with the fruits, 11,650 DEGs were observed in the petals, with 6413 upregulated and 5237 downregulated (Figure 4A). Similarly, in a comparison of petals with roots, 12,289 DEGs were observed, with 7221 upregulated and 5068 downregulated (Figure 4B). In a further investigation into the functions of DEGs, gene ontology (GO) enrichment analysis was conducted to reveal the activation or deactivation of distinct gene functions. Several DEGs were enriched in carbon–oxygen lyase activity and isoprenoid metabolic processes when comparing petals to fruits (Figure 4C). When comparing petals to roots, the DEGs associated with carbon–oxygen lyase activity, hydrocarbon metabolic processes, isoprenoid metabolic processes, and terpene metabolic processes were found to be accumulated (Figure 4D). These DEGs are believed to play a role in terpenoid metabolite metabolism, aligning with their tissue specificity in *R. rugosa*.

### 2.3. Integrative Analysis of Transcriptome and Metabolome

**Figure 5:**
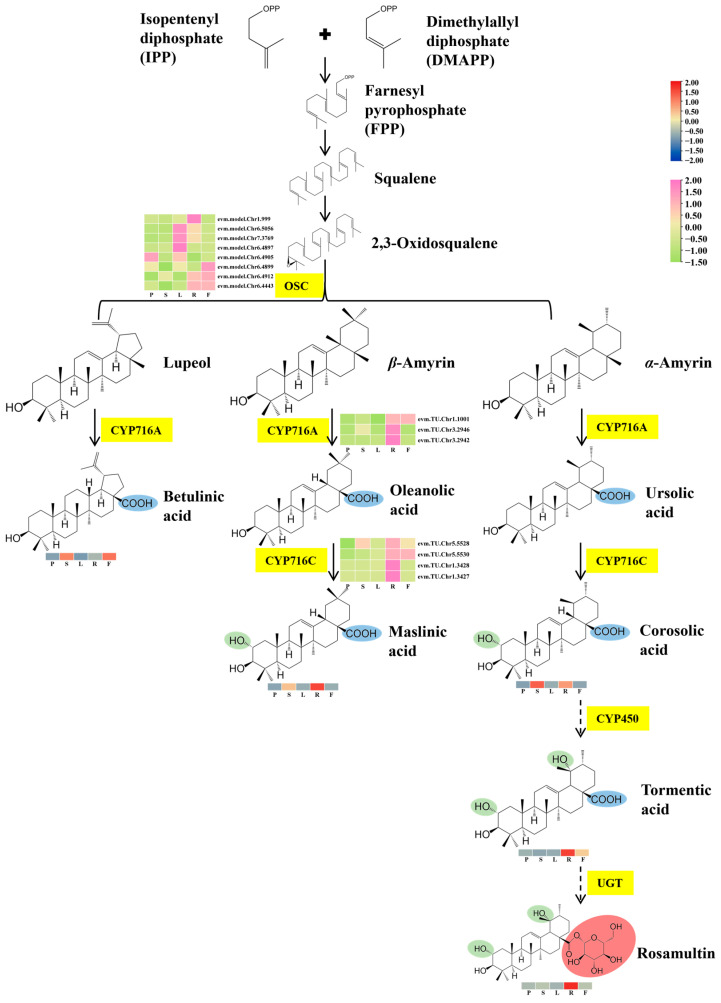
Changes in gene expression and metabolite accumulation levels of triterpene metabolic pathway. Yellow markings indicate genes, while heatmaps display key metabolites and transcripts associated with triterpenoid biosynthesis in various tissues.

The DEGs were selected for a metabolite–gene correlation analysis, as depicted in (Supplementary Figure S4). The integration of transcriptome and metabolome analysis resulted in the identification of functional genes in the triterpene metabolic pathway, as demonstrated in (Figure 5). The findings indicated that the roots and fruits were found to have a higher accumulation of triterpenoids, such as maslinic acid, betulinic acid, tormentic acid, and rosamultin. Additionally, key genes that showed a high expression level in roots and fruits were identified, indicating that they were potentially involved in triterpene biosynthesis. Specifically, four *OSC* genes (evm.TU.Chr1.999, evm.TU.Chr6.4899, evm.TU.Chr6.4912, and evm.TU.Chr6.4443) were identified to potentially facilitate the biosynthesis of triterpene scaffolds from 2,3-oxidosqualene. Additionally, three *CYP716A* genes (evm.TU.Chr1.1001, evm.TU.Chr3.2946, and evm.TU.Chr3.2942) and four *CYP716C* genes (evm.TU.Chr5.5528, evm.TU.Chr5.5530, evm.TU.Chr1.3428, and evm.TU.Chr1.3427) were found that are carboxylated at the C-28 position and hydroxylated at the C-2 position. The diverse accumulation patterns of these compounds might have resulted from variations in the gene expression patterns.

### 2.4. Characterization of RrOSC10 

**Figure 6:**
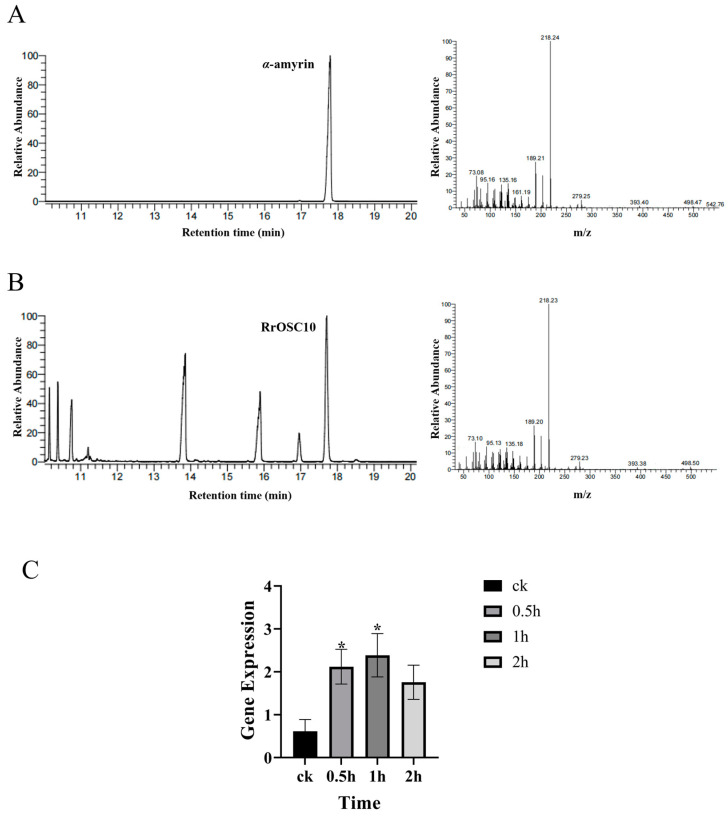
Characterization of *RrOSC10*. (**A**) The standard compound *α*-amyrin is shown in the GC-MS chromatograms. (**B**) The major triterpenes extracted from yeast expressed with *RrOSC10* are shown in the GC-MS chromatograms. (**C**) Gene expression analysis of *RrOSC10* under salt treatment. *, *p* < 0.05.

The OSC enzyme, due to its engagement in the first step of the triterpene biosynthesis pathway, was further characterized. A prior phylogenetic analysis indicated that out of the 8 *OSC* genes, only two *OSC* genes, *RrOSC6* (evm.TU.Chr6.4443) and *RrOSC10* (evm.TU.Chr6.4912), could be potential candidate genes for *α*-amyrin [30]. The protein sequence identity between RrOSC6 and *RrOSC10* was 86%. To further study the catalytic activities of RrOSC6 and *RrOSC10*, the full-length coding regions were cloned into a yeast expression vector, pESC-Leu, and subsequently transformed into a yeast, AM94, respectively. After cultivation, the products were extracted from the yeasts and identified using gas chromatography–mass spectrometry (GC–MS). The results showed that yeast cells expressing *RrOSC10* accumulated *α*-amyrin as a single product (Figure 6A), whereas the yeast cells containing RrOSC6 did not produce any product. This was confirmed by comparing it with authentic standards.

Terpenoids play a significant role in mediating the interaction between plants and their environment [16,36]. To investigate *R. rugosa*’s response to salt stress, *R. rugosa* plants were treated with salt, and the expression of the *RrOSC10* gene was analyzed using RT-qPCR. The results indicated a significant increase in the mRNA levels of *RrOSC10* in the roots of *R. rugosa* at 0.5 h and 1 h, followed by a decrease at 2 h. This fluctuating pattern suggested a potential defensive function of *RrOSC10* in response to salt-induced stress (Figure 6B).

## 3. Discussion

Metabolomics is a powerful technique for comprehensively profiling and comparing the diverse secondary metabolites associated with each biological phenotype through high-throughput characterization [37]. This approach enables the detection of many secondary metabolites or their derivatives under normal conditions or in response to various environmental stimuli [38]. Our research revealed a diverse range of terpenoid compounds and provided new insights into the previously unexplored terpenoid composition in *R. rugosa*. This approach enhanced the understanding of *R. rugosa*’s chemical profile and established a foundation for future investigations into the medicinal significance of these non-volatile terpenes. Integrating omics data analysis, such as transcriptomics and proteomics, is beneficial for a comprehensive understanding of the biochemical landscape [39,40]. This study thoroughly investigated the non-volatile terpenoid profile in various tissues of *R. rugosa*, offering insightful observations on the distribution and diversity of these secondary metabolites in the plant. The terpenoids displayed distinct patterns, significantly varying their levels and compositions across diverse plant organs. In particular, the fruits and roots showed significant differences compared to the petal, leaf, and stamen tissues. Furthermore, the K-means cluster analysis suggested that specialized biosynthetic pathways or ecological roles are associated with specific tissue types.

Triterpenoids exhibit a wide array of pharmacodynamic functions, encompassing diverse roles, including anti-inflammatory properties [41], antioxidant effects [42], anticancer potential [43], immunomodulatory properties [44], antimicrobial effects, hepatoprotective actions [45], and neuroprotective properties [46]. In this study, Asiatic acid, annurcoic acid, maslinic acid, pomolic acid, rosamultic acid, and ursonic acid were identified in *R. rugosa.* These compounds have been reported in roses and other species and are associated with medicinal value [47,48]. 

Three groups of triterpenoids (ursane-, oleanane-, and lupane-type) were identified in the fruit of *R. rugosa*, indicating substantial variances in their biosynthesis and accumulation patterns [49]. The fruit samples contained all three types of triterpenes, with a notably higher accumulation of compounds from subclades 4 and 9, suggesting a unique origin or biosynthetic pathway specific to fruits. Through NMR analysis, the methanol extract derived from the roots of *R. rugosa* enabled the identification of 13 triterpenoid saponins, which demonstrated an association with rat intestinal sucrase inhibitors [50]. However, two triterpenoid saponins (rosamultin and 2,3,19-trihydroxyurs-12-en-23,28-dioic acid-28-O-glucoside) were detected in the roots in this study. The observed variations may be attributed to differences in measurement methods. Furthermore, no previous studies have investigated the triterpenoid profiles of petals, leaves, and stamen. Therefore, this study represents the first exploration of these profiles within these specific tissues, providing a foundation for understanding their diverse properties.

The combined omics data have successfully been used to investigate the complex metabolic network [51]. In this study, the integrative analysis of triterpene metabolites and transcriptomics were obtained. Additionally, the expression patterns of all genes were analyzed, and these data will benefit future studies on *R. rugosa*. For example, this analysis can also quickly identify candidate modification enzymes like P450 and UGT.

*R. rugosa* plants are known for their wide adaptability to various environments, which may be associated with the presence of triterpenoids. In this study, the integration of metabolomics, transcriptomics, and biochemical approaches led to the identification of an *α*-amyrin synthase responsible for triterpene biosynthesis in *R. rugosa*. A previous study identified a lupeol synthase (RrOSC12: evm.TU.Chr1.999) from *R. rugosa* through in vitro analysis [30]. The transcriptome data revealed that this gene was primarily expressed in the roots, whereas betulinic acid accumulation was higher in the stamens and fruits, suggesting an essential role of CYP716A enzymes in these tissues. The *α*-amyrin synthases have been identified in *Eriobotrya japonica* (EjAS) and *Malus × domestica* as catalysts that convert 2,3-oxidosqualene into *α*-amyrin as the main product, along with the minor products *β*-amyrin and lupeol [33,52]. A biochemical analysis has shown that *α*-amyrin is the primary product, although in relatively small quantities. This indicated a decrease in *RrOSC10* enzymatic activity in the yeast system, emphasizing the need for further optimization to achieve comprehensive characterization. In contrast, RrOSC6 did not produce any product in the yeast system. This may be due to its ineffective expression in the yeast system. The tandem duplication of RrOSC6 and *RrOSC10* on chromosome 2 indicates divergent evolution [26]. These findings strongly suggested the involvement of these synthases in triterpene synthesis. Moreover, the findings revealed a notable increase in *RrOSC10* gene expression in the roots of *R. rugosa* 0.5 h after salt treatment. It demonstrated that *RrOSC10* played an active role in the initial phase of the plant’s defense against induced salt stress. The subsequent decrease observed at 2 h post-treatment suggested that the plant likely regulated *RrOSC10* expression through feedback after the initial response. This biochemical evidence has provoked further investigation into the potential additional functions of RrOSCs. The production of avenacins in roots by the oat *β*-amyrin synthase AsbAS1 provided resistance against various fungal pathogens [53]. It would be interesting to investigate the potential role of *RrOSC10* below the ground. The rice gene OsOSC12/OsPTS1 has a specific expression in anthers and is responsible for disrupting pollen coat formation. This results in humidity-sensitive male sterility [54]. The dataset included measurements from stamen tissues, which provide a base for future research investigations of stamen-specific OSCs. *Artemisia annua* uses OSC2 and CYP716A14V2 enzymes to produce specific triterpenoids in its cuticle wax layer, which protect against both biotic and abiotic stress [55]. *Salvia mirzayanii* plants exhibited elevated levels of oleanolate 3-*β*-d-glucuronoside-28-glucoside, taxol, and glycyrrhetinate terpenoids when subjected to salt stress [56]. Elucidating the function of RrOCS10 and the integrative analysis of CYP450s and UGTs can help us better understanding the complex molecular mechanisms underlying triterpene biosynthesis in *R. rugosa* [57,58]. 

## 4. Materials and Methods

### 4.1. Materials and Reagents

The experimental *R. rugosa* plants were cultivated at Yangzhou University in Yangzhou, China at 32.391° N, 119.419° E. The samples used for this study were collected in May 2022 from the following five different parts: petals (P), leaves (L), stamens (S), roots (R), and fruits (F). The experiment utilized 15 samples of *R. rugosa*, each with three biological replicates. All samples were rapidly frozen in liquid nitrogen after collection and stored in at −80 °C. The compound *α*-amyrin was purchased from Sigma-Aldrich^®^ (www.sigmaaldrich.cn (accessed on 6 May 2023), Merck, Darmstadt, Germany). 

### 4.2. Metabolite Extraction and Identification

After collecting the samples, they were submitted to Metware Biotechnology Co., Ltd. (Wuhan, China) for qualitative and quantitative analyses of the terpene compounds. The samples were freeze-dried, powdered, and weighed (50 mg per sample). Then, 1.2 mL solution of 70% methanol extract was added for rotational evaporation. Then, the mixture was filtered for 3 min at 12,000 rpm and analyzed using a UPLC–ESI–MS/MS system (UPLC, ExionLC™ AD, https://sciex.com.cn/ (accessed on 10 March 2023); MS, Applied Biosystems 6500 Q TRAP, https://sciex.com.cn/ (accessed on 10 March 2023)). Agilent SB-C18 1.8 µm, 2.1 mm × 100 mm, was used in the UPLC and ESI experimental conditions. The mobile phase consisted of ultrapure water (with 0.1% formic acid) as Phase A and acetonitrile (with 0.1% formic acid) as Phase B in the chromatographic column. An elution gradient was utilized. The process started with 5% B at 0.00 min, and the proportion of B increased linearly to 95% within 9.00 min and remained at 95% for 1 min. From 10.00 to 11.10 min, the proportion of B was decreased to 5% and kept constant until 14.00 min. The column’s temperature was maintained at 40 °C, the flow rate was 0.35 mL/min, and 2 μL of the sample was injected. The electrospray ionization (ESI) temperature was adjusted to 500 °C. In positive ion mode, the ion spray voltage (IS) was 5500 V, while in negative ion mode, it was −4500 V. Gas source I (GSI), gas source II (GSII), and curtain gas (CUR) were set at 50, 60, and 25 psi, respectively. Collision-induced dissociation parameters were set to high. A triple quadrupole (QQQ) scan was carried out using the multiple reaction monitoring (MRM) mode. Nitrogen was used as the collision gas with medium settings. After certain voltage adjustments, each MRM ion pair was optimized by adjusting the collision energy (CE) and declustering potential (DP). A specific set of MRM ion pairs was monitored to capture metabolites during each elution period.

### 4.3. Screening for Differential Metabolites

The statistical function prcomp in R 4.2.3 (www.r-project.org (accessed on 20 March 2023)) was utilized to conduct a principal component analysis (PCA). The R software calculated the Pearson correlation coefficient (PCC) between samples. Differential metabolites between the two groups were identified using an absolute Log_2_FC (|Log_2_FC| ≥ 1.0). Subsequently, quantitative and qualitative analyses of the identified metabolites, along with sample grouping, and heatmaps were generated. The relative levels of the various metabolites in all comparison groups were normalized using unit variance scaling (UV) and then subjected to a K-means cluster analysis.

### 4.4. Transcriptome Analysis

The cDNA libraries were sequenced using the Illumina sequencing platform by Metware Biotechnology Co., Ltd. in Wuhan, China. RNA quality and integrity were assessed using the NanoPhotometer spectrophotometer (IMPLEN, Westlake Village, CA, USA) and Agilent 2100 bioanalyzer (Agilent Technologies, Santa Clara, CA, USA). Library construction was carried out using the NEBNext^®^ UltraTM RNA Library Prep Kit (NEB, Ipswich, MA, USA). Simultaneously, Oligo(dT) and magnetic beads enriched with mRNA containing a polyA tail. The M-MuLV reverse transcriptase system was utilized to synthesize the first cDNA strand, while the second cDNA strand was synthesized in the DNA polymerase I system using dNTPs.

### 4.5. Identification of Differentially Expressed Genes

Gene alignment was assessed utilizing FeatureCounts version 1.6.2, and the fragments per kilobase of exon model per million mapped fragments (FPKM) were calculated for each gene. A differential expression analysis between the two groups was executed using DESeq2 version 1.22.1, with adjustment of *p* values using the Benjamini and Hochberg method. The volcano plot illustrated the threshold for significant differential expression using |log2foldchange|. A GO enrichment analysis of differentially expressed genes (DEGs) in petals versus roots and petals versus fruit was performed utilizing GO terms GO:0010333 and GO:0042214. 

### 4.6. Integrated Analysis of Transcriptome and Metabolome

The intricate relationship between metabolism and genes across different parts of the rose plant was investigated through a comprehensive analysis of transcriptome and metabolome data. OSCs were identified using the N-terminal domain (PF13249) and C-terminal domain (PF13243) with HMMER and aligned with the sequences [30]. CYP716As were aligned with the typical sequence of *Medicago truncatula* CYP716A12 (Accession DQ335781), and CYP716Cs were aligned with CaCYP716C11 (accession KU878852.1) [59]. The screened key genes were involved in the biosynthesis of terpenoid metabolites. 

### 4.7. Enzymatic Assays in Yeast 

The coding sequences of RrOSCs were initially sub-cloned into the pESC-leu yeast expression vector, followed by transformation into yeast strain AM94. To analyze triterpenoid products, yeast cultures expressing RrOSCs were collected, and the culture extracts were derivatized using trimethylsilylating agents. The derivatized samples were subsequently analyzed using gas chromatography–mass spectrometry (GC–MS) analysis (7890A-7697A).

### 4.8. Salt Treatment and Quantitative Real-Time PCR (RT-qPCR) 

The roots of two-month-old *R. rugosa* seedlings were immersed in a 170 mM NaCl solution, while water was used as the control. Samples were extracted at intervals of 0.5, 1, and 2 h after treatment [60,61]. Both NaCl-treated and control samples were subjected to RNA extraction using a kit, and cDNA was reverse-transcribed using the HiScript II Q Select RT SuperMix for qPCR kit (Vazyme, Nanjing, China). The RT-qPCR analysis utilized the SYBR Green qPCR Master Mix kit (Vazyme, Nanjing, China), and the resulting data were evaluated through a relative quantitative method before being visualized using GraphPad Prism 9 (https://www.graphpad.com/ (accessed on 1 August 2023)).

## 5. Conclusions

This study demonstrates the complex mechanism underlying the biosynthesis and accumulation of triterpenoids in *R. rugosa* through the integrated analysis of metabolomics and transcriptomics. First, the dynamic accumulation of triterpenoids in different tissues was observed, particularly noting diverse triterpenoid profiles in the roots and fruits. Second, this combined analysis identified the *ROSC10* gene encoding OSC, which plays a pivotal role in α-amyrin biosynthesis. The upregulation of *RrOSC10* in response to salt stress suggested its involvement in salinity tolerance. This study increased our understanding of the plant’s medicinal potential and established the foundation for future research to explore its beneficial properties for different purposes. 

## Figures and Tables

**Table 1:** Summary of RNA-Seq data of five issues of *R. rugosa*.

Sample	Raw Reads (bp)	Clean Reads (bp)	Clean Base (Gb)	Error Rate (%)	Q20 (%)	Q30 (%)	GC Content (%)
P	83,693,494	81,511,490	12.23	0.03	97.78	93.58	46.52
L	55,959,736	54,116,102	8.12	0.03	97.90	93.86	47.46
S	66,390,516	64,241,230	9.64	0.03	97.71	93.43	47.34
R	66,390,338	64,211,898	9.63	0.03	97.85	93.72	46.91
F	63,512,398	60,685,890	9.10	0.03	97.83	93.73	47.17
Average	67,189,296	64,953,322	9.744	0.03	97.81	93.66	47.08

## Data Availability

Data are contained within the article and the Supplementary Materials.

## Supplementary Materials

The following supporting information can be downloaded at https://www.mdpi.com/article/10.3390/plants13121600/s1: Figure S1: Mass spectrometry detection of total ion current plots; Figure S2: Sample-to-sample correlation analysis between different tissues; Figure S3: Classification of metabolites and tissue variations. Figure S4: Differential gene and metabolite-related clustering heatmap in petal versus root; Figure S5: Differential gene and metabolite-related clustering heatmap in petal versus fruit; Table S1: Total terpenoids were identified; Table S2: Expression patterns of 29,758 genes.
