# Supplementary material for: Integrated Metabolomics and Transcriptomics Analysis Reveals New Insights into Triterpene Biosynthesis in Rosa rugosa
Source: Plants (Basel). 2024 Jun 8;13(12):1600. doi: 10.3390/plants13121600 (PMC11207392; doi:10.3390/plants13121600)
Supplement: Supplementary file 1 [file plants-13-01600-s001.zip › Supplementary Figure0516.pptx]

## Slide 1
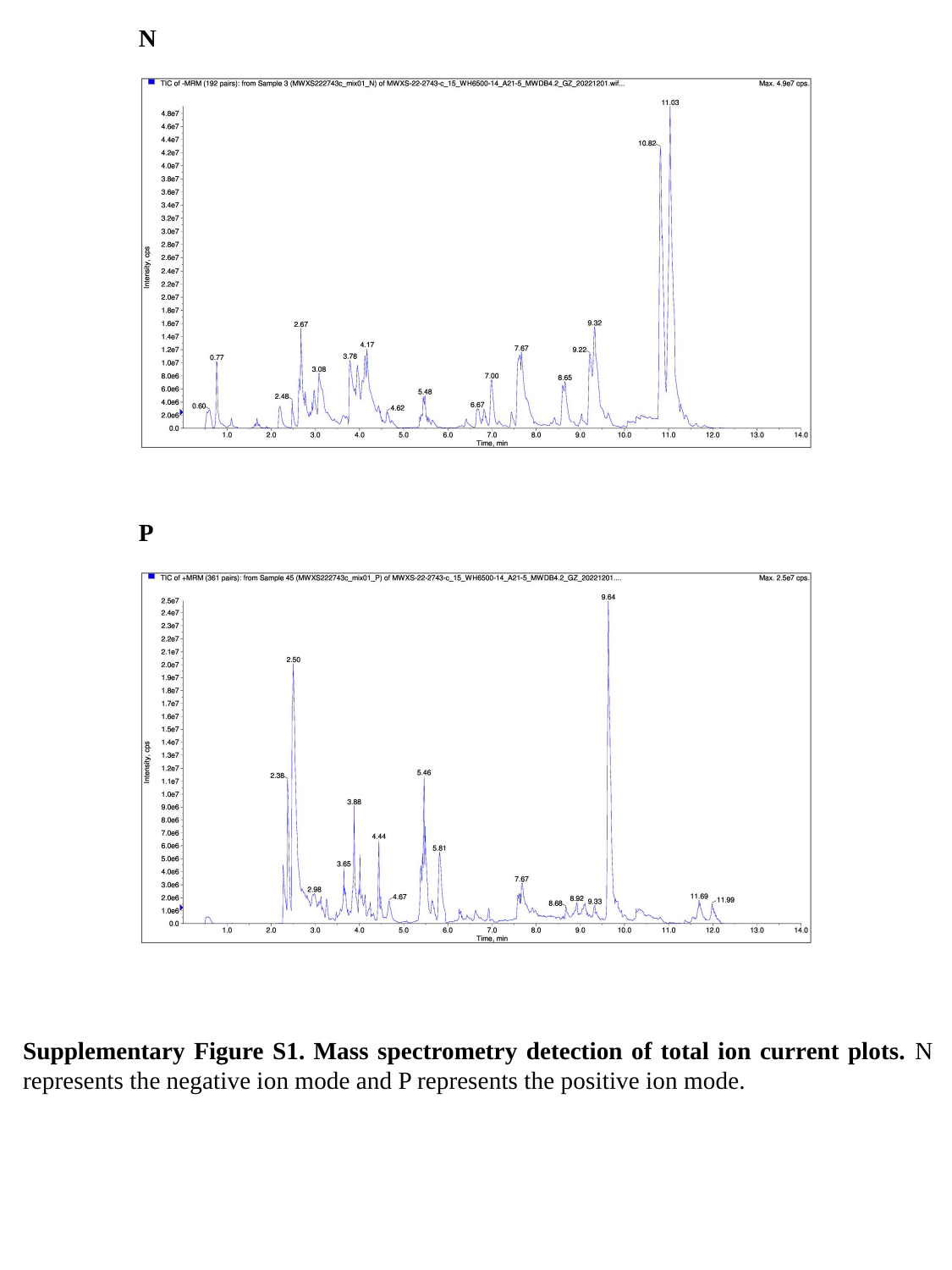

N
P
Supplementary Figure S1. Mass spectrometry detection of total ion current plots. N represents the negative ion mode and P represents the positive ion mode.

## Slide 2
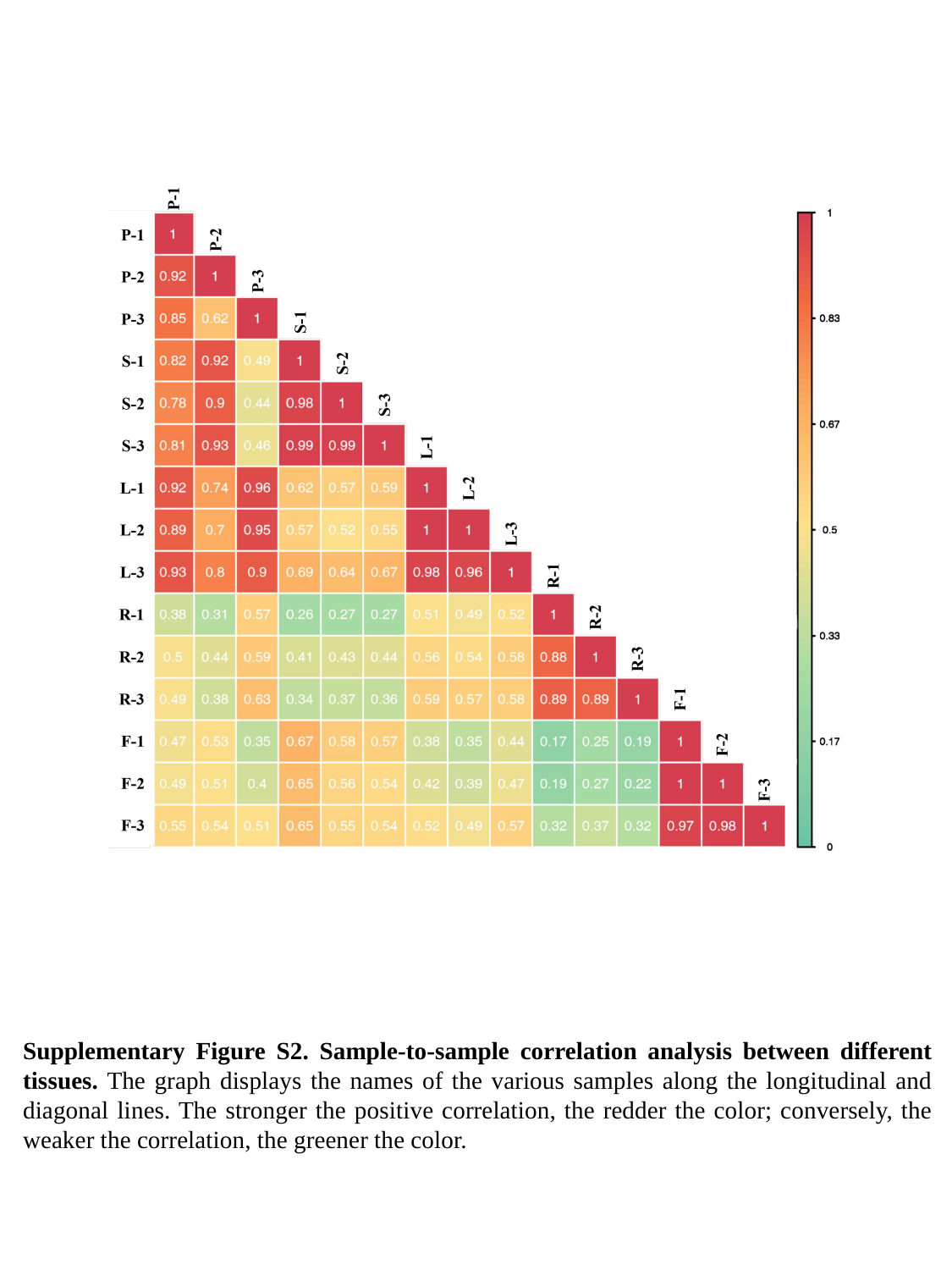

Supplementary Figure S2. Sample-to-sample correlation analysis between different tissues. The graph displays the names of the various samples along the longitudinal and diagonal lines. The stronger the positive correlation, the redder the color; conversely, the weaker the correlation, the greener the color.

## Slide 3
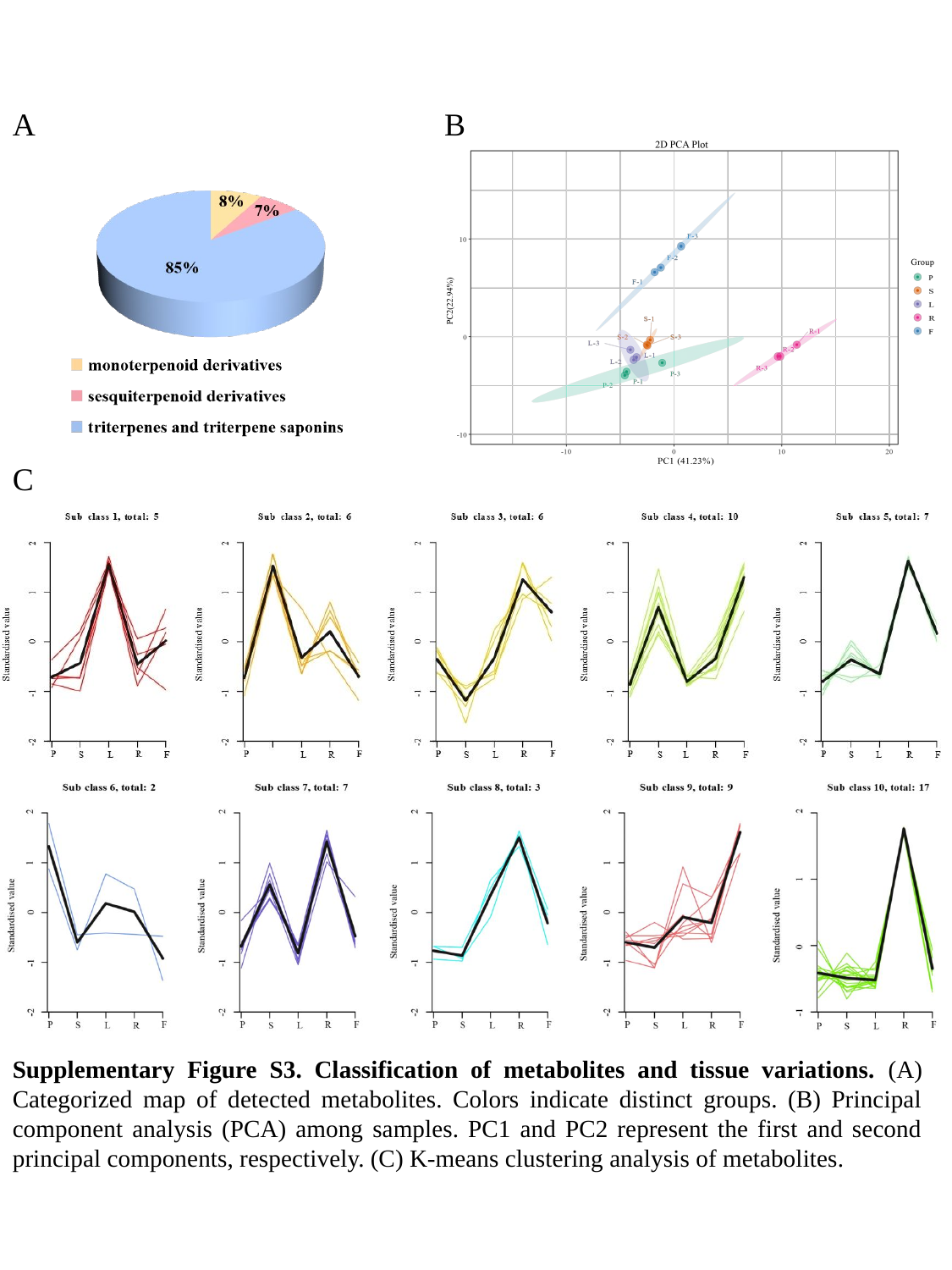

A
B
C
Supplementary Figure S3. Classification of metabolites and tissue variations. (A) Categorized map of detected metabolites. Colors indicate distinct groups. (B) Principal component analysis (PCA) among samples. PC1 and PC2 represent the first and second principal components, respectively. (C) K-means clustering analysis of metabolites.

## Slide 4
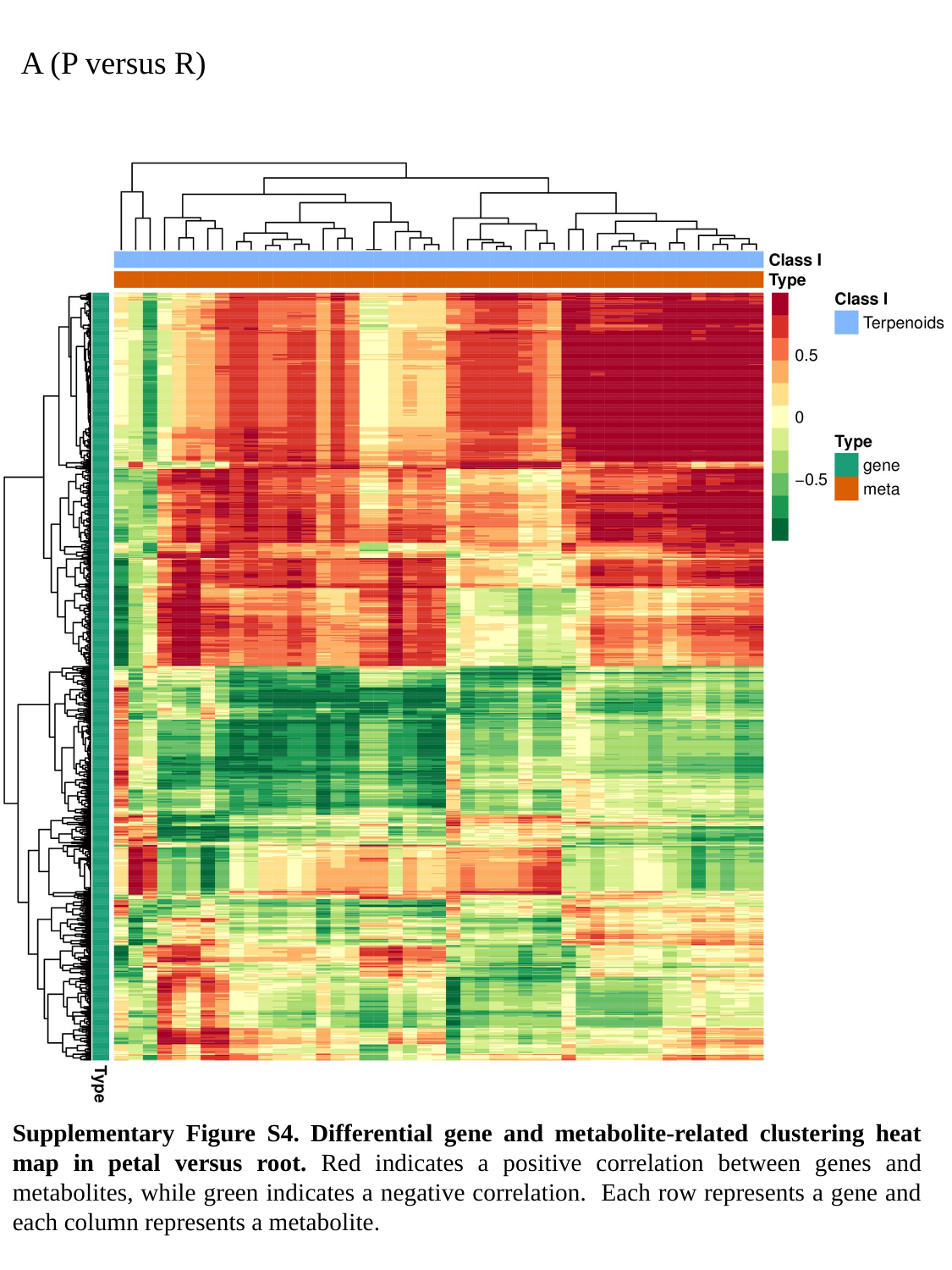

A (P versus R)
Supplementary Figure S4. Differential gene and metabolite-related clustering heat map in petal versus root. Red indicates a positive correlation between genes and metabolites, while green indicates a negative correlation. Each row represents a gene and each column represents a metabolite.

## Slide 5
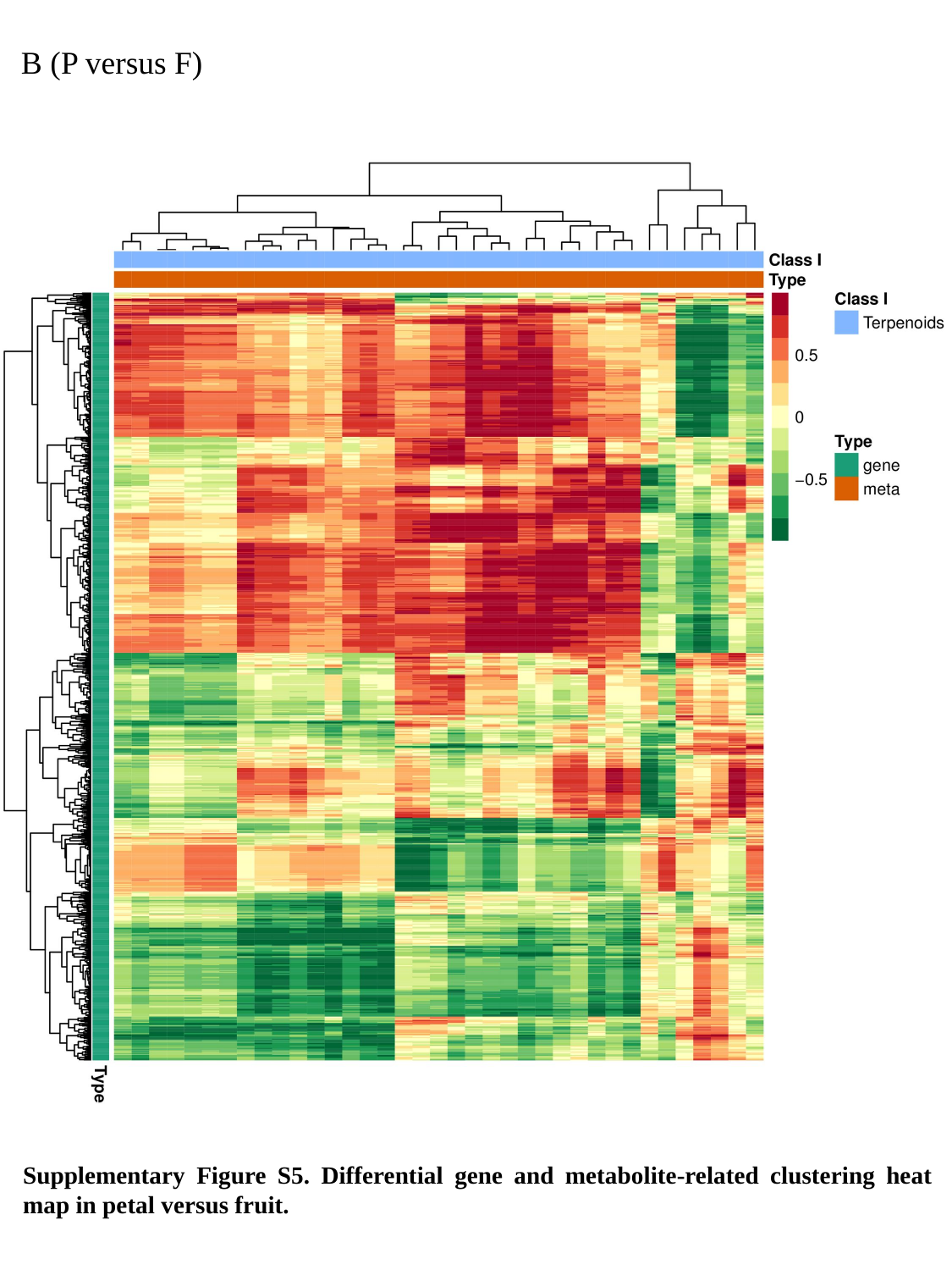

B (P versus F)
Supplementary Figure S5. Differential gene and metabolite-related clustering heat map in petal versus fruit.
